# Phytochemical Composition, *In Vitro* Antimicrobial, Antioxidant, and Enzyme Inhibition Activities, and *In Silico* Molecular Docking and Dynamics Simulations of *Centaurea lycaonica*: A Computational and Experimental Approach

**DOI:** 10.1021/acsomega.3c01819

**Published:** 2023-06-13

**Authors:** Hanifa Fatullayev, Leyla Paşayeva, Ismail Celik, Ufuk İnce, Osman Tugay

**Affiliations:** † Department of Pharmaceutical Chemistry, Faculty of Pharmacy, Erciyes University, Kayseri 38039, Türkiye; ‡ Department of Pharmacognosy, Faculty of Pharmacy, Erciyes University, Kayseri 38039, Türkiye; § Department of Pharmaceutical Microbiology, Faculty of Pharmacy, Erciyes University, Kayseri 38039, Türkiye; ∥ Department of Pharmaceutical Botany, Faculty of Pharmacy, Selçuk University, Konya 42130, Türkiye

## Abstract

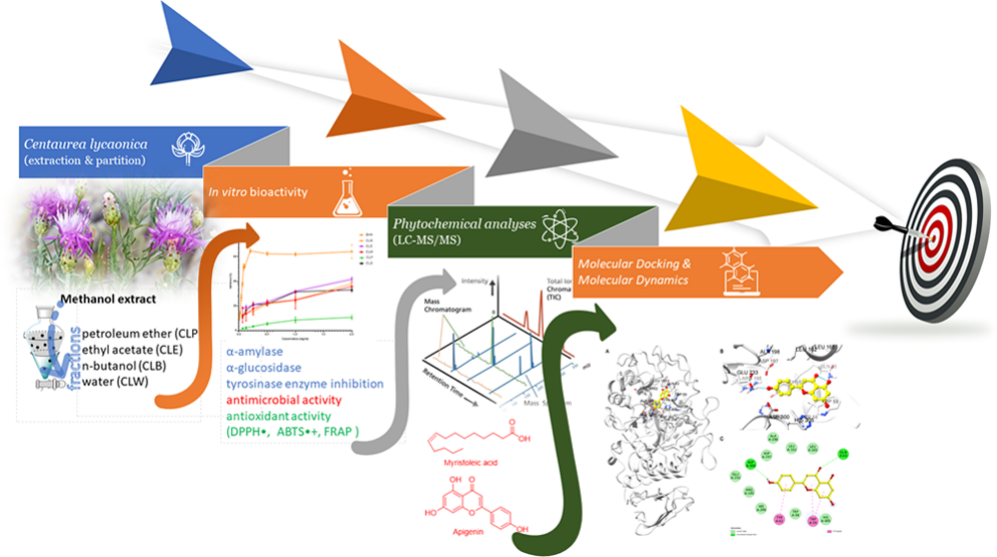

*Centaurea lycaonica* is a local endemic species from the *Centaurea* L. genus. The *Centaurea* species has a wide range of usage in treating diseases in folk medicine. There are limited biological activity studies on this species in the literature. This study investigated enzyme inhibition and antimicrobial activity, antioxidant effect, and chemical content of extract and fractions of *C. lycaonica*. Enzyme inhibition activity was tested by α-amylase, α-glucosidase, and tyrosinase enzyme inhibition methods and antimicrobial activity by the microdilution method. The antioxidant activity was investigated using DPPH^•^, ABTS^•+^, and FRAP tests. The chemical content was determined by LC-MS/MS. The methanol extract showed the highest activity for α-glucosidase and α-amylase, even surpassing the positive control acarbose, with IC_50_ values of 56.333 ± 0.986 and 172.800 ± 0.816 μg/mL, respectively. Additionally, the ethyl acetate fraction also exhibited high activity for α-amylase with an IC_50_ value of 204.067 ± 1.739 μg/mL and tyrosinase with an IC_50_ value of 213.900 ± 1.553 μg/mL. Moreover, this extract and fraction were found to have the highest total phenolic and flavonoid contents and antioxidant activity. Additionally, LC-MS/MS analyses of active extract and fraction revealed mainly the presence of phenolic compounds and flavonoids. *In silico* molecular docking and molecular dynamics simulation studies of determining compounds apigenin and myristoleic acid, common in CLM and CLE extracts and active against α-glucosidase and α-amylase, were performed. In conclusion, methanol extract and ethyl acetate fraction showed potential enzyme inhibition and antioxidant activity as a natural agent. Molecular modeling studies corroborate the findings of *in vitro* activity analyses.

## 1 Introduction

The use of medicinal plants in the treatment and prevention of diseases is becoming increasingly common. The main reasons are the safe side-effect and interaction profile of medicinal plants and natural agents compared to synthetic ones, easy accessibility, and wide therapeutic application range. Researchers have focused on using medicinal plants to treat diseases in recent years. Many drugs used today have been synthesized, inspired by bioactive compounds in plants.^1^ Free radicals cause hypertension, diabetes, cancer, and hyperpigmentation. As a more specific subgroup of free radicals, the damage caused by reactive oxygen species (ROS) to cellular structures is prevented by scavenging them with antioxidants. There is a balance between the antioxidant system and free radicals. Plants are rich sources of natural antioxidants, which are preferred because of their safety and low toxicity.^2^

**Figure 1:**
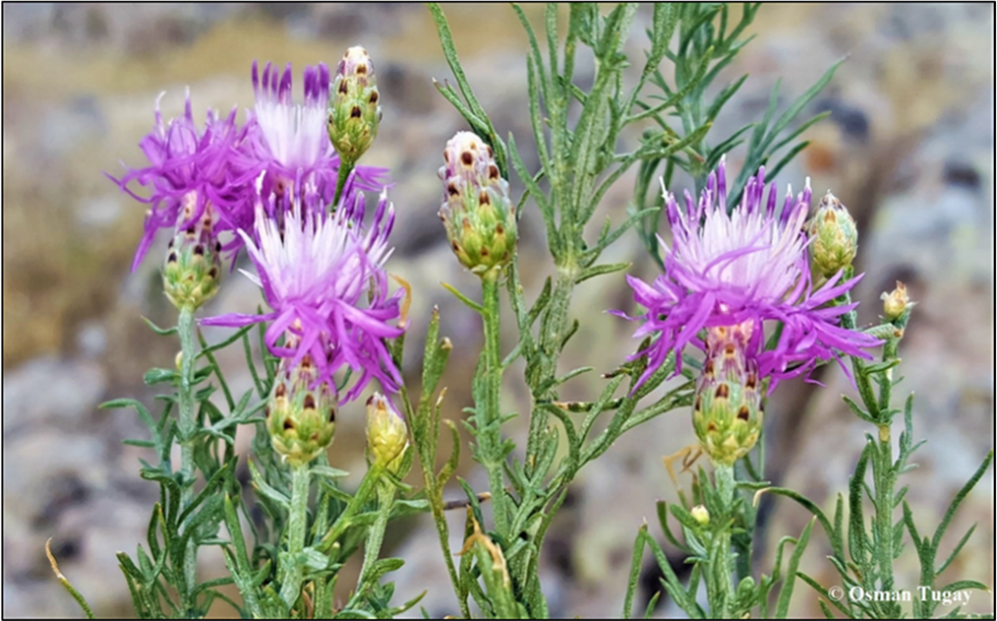
General view of *Centaurea lycaonica* Boiss. & Heldr (Photo: by Osman Tugay).

The *Centaurea lycaonica* Boiss. & Heldr. species is included together with *C. amaena* Boiss. & Bal., *C. aphrodisea* Boiss., *C. cadmea* Boiss., *C. hierapolitana* Boiss., *C. luschaniana* Heimerl ex Stapf, *C. lycia* Boiss., *C. tossiensis* Freyn & Sint., and *C. wagenitzii* Hub.-Mor. species in Sect. Phalolepis (Cass.) DC. of *Centaurea* L. genus. The general characteristics of Sect. Phalolepis can be given as follows: Appendages almost orbicular, hyaline with a firmer center, entire or irregularly lacerate, ending in a short mucro or spinule, decurrent or not decurrent.^3^*C. lycaonica*, known as “Zarif düğme”,^4^ is perennial, stems to 30 cm, simple or with one branch, lower leaves pinnatipartite with linear segments, c. 1 mm broad, sparsely arachnoid above, upper leaves simple, similar to segments of lower Figure 1. Involucre 10–11 × 5–6 mm, appendages large, almost concealing basal part of phyllaries, almost circular, c. 3 mm broad, with a dark brown, firm central part, and broad minutely denticulate and usually lacerate hyaline, shortly decurrent margin, emarginate at the tip, terminal mucro absent or minute. Flowers are rose-purple, marginal, scarcely radiant. Achenes are 2.5–3.5 mm and pappus is 3 mm. Its flowering time is from June to July, and it grows on open hillsides in short turf, sparse coniferous woods, and scrub at an elevation of 1570–1580 m. *C. lycaonica* is distributed only in Konya (Central Anatolia) and has an Irano-Turanian element. The species is morphologically close to *C. luschaniana*, differing primarily by having the plant nearly glabrous and terminal segment of basal leaves 1–2 mm broad.^3^

Molecular docking studies are an essential computational method in modern drug research.^5^ This modeling method allows simulating and predicting the interactions of especially small molecular weight compounds with the target macromolecules such as proteins, enzymes, RNA, and DNA.^6^ Molecular dynamics (MD) simulations are frequently used in drug designs to explain the interactions, stability, and dynamical changes of protein–ligand complexes obtained from molecular docking.^7^

**Figure 2:**
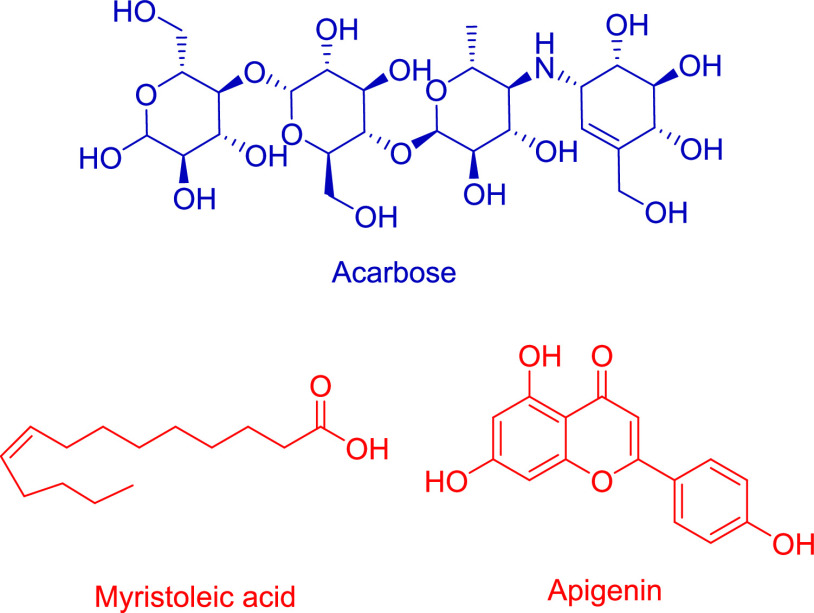
Chemical structure of acarbose and common compounds of methanol extract and active fraction.

Based on the available literature, no prior studies have investigated the phytochemical and biological activities of the *C. lycaonica* species. Therefore, this study aimed at investigating the potential α-amylase, α-glucosidase, and tyrosinase inhibitory effects, as well as the antimicrobial activities, antioxidant potential (measured through DPPH, ABTS, and FRAP tests), and phytochemical analyses of the active extract/fraction (using LC-MS/MS). Apigenin and myristoleic acid were discovered to be common compounds in methanol extract and an active fraction (Figure 2).

## 2 Materials and Methods

### 2.1 Plant Material

The aerial parts of *C. lycaonica* were harvested from the area of Konya province in Türkiye and identified by Prof. Dr. Osman Tugay (O.Tugay 16.843 & C. Ceylan).

### 2.2 Extraction of the Plant Material

The plant material weighing 600 g was ground after drying. Maceration with methanol (3 × 24 h) was employed to prepare the extract. The methanol extract (CLM) was subsequently fractionated using solvents with varying polarities. As a result, petroleum ether (CLP), ethyl acetate (CLE), *n*-butanol (CLB), and water (CLW) fractions were obtained. At the end of extraction, all extract and fractions evaporated in vacuo (37 °C), and then lyophilized.

### 2.3 *In Vitro* Studies

#### 2.3.1 Enzyme Inhibition Assays

To study the inhibition activity of α-amylase and α-glucosidase,^8^ the extract and fractions were diluted to 40–2000 μg/mL concentrations. Acarbose was used as a positive control during the assay. The absorbance of the samples was read at 540 nm for the α-amylase assay and at 400 nm for the α-glucosidase assay. The inhibitory activity of both enzymes was calculated using eq (1)
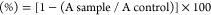
1The tyrosinase inhibitory activity assay was performed at 492 nm using a microplate reader.^9^ According to the method, 20 μL of various concentrations of extract and fractions, 20 μL of enzyme solution (250 U/mL), and 100 μL of phosphate buffer (0.1 M, pH 6.8) were mixed in a 96-well plate. After 10 min of incubation, 20 μL of l-tyrosine solution (3 mM) was added and incubated for 30 min at 25 °C. Kojic acid was used as a positive control in this assay.

#### 2.3.2 Total Phenolic (TPC) and Total Flavonoid (TFC) Contents

In this research, the TPC and the TFC were determined with slight modification of the method of Zengin et al.^10^ The TPC results were calculated as gallic acid equivalents (GAE) per gram of extract. The TFC values as milligrams of catechin equivalent.

#### 2.3.3 Antioxidant Activity Assays

In the DPPH^•^ scavenging activity assay, different concentrations of extract and fractions were mixed with 0.05 M Tris-HCl buffer and DPPH solution.^11^ This research used butylated hydroxyanisole (BHA) as a positive control.

The ABTS^+•^ scavenging capacity of the extract and fractions was determined as the Trolox equivalent. ABTS solution was diluted with methanol to an absorbance of 0.700 ± 0.02 at 734 nm to begin the assay. Then the sample solution was added to the ABTS solution, and the color change was recorded.^12^

In the FRAP assay, the FRAP values of extract and fractions were determined at 2, 1, and 0,5 mg/mL concentrations.^11^ In this assay, the change of color was recorded at 593 nm. The results were expressed as mmol of Fe^2+^ equivalents per g of extract/fraction weight (mmol Fe^2+^ /g).

#### 2.3.4 Antimicrobial Activity

In this study, the sensitivity of *Staphylococcus aureus* ATCC 29213, *Escherichia coli* ATCC 25922 (Gram-positive and Gram-negative bacteria, respectively), *Candida albicans* ATCC 10231 (fungus), and the clinical isolates of these microorganisms to plant extract and fractions were tested according to the method of Paşayeva et al.^11^ The study was conducted according to the Clinical Laboratory Standards Institute (CLSI) M100-S28 protocol for bacteria^13^ and the CLSI M27-A3 protocol for fungi.^14^

### 2.4 Phytochemical Analysis

The phytochemical compositions of active methanol extract and the ethyl acetate fraction were determined by LC-MS/MS. The CLM and CLE stock solution was prepared in methanol at 10 μg/mL concentration. The samples were directly injected in LC/MS-MS at a 0.5 mL/min flow rate and an injection volume of 1 μL. The mobile phase was a mixture of acetonitrile (A) and methanol/formic acid (99:1, v/v) (B) as 80% solvent A and 20% solvent B.

### 2.5 Statistical Analysis

Statistical analysis was performed using GraphPad Prism Software (La Jolla, CA). Statistically significant values were compared using one-way ANOVA with Tukey’s post hoc test, and *p* values of less than 0.05 were considered statistically significant.

### 2.6 *In Silico* Studies

#### 2.6.1 Molecular Docking

Molecular docking was performed using the AutoDock Vina-based^15^ CB-Dock2 server (https://cadd.labshare.cn/cb-dock2/php/index.php).^16^ The compounds apigenin (PubChem ID: 5280443), myristoleic acid (PubChem ID: 5281119), and standard acarbose (PubChem ID: 41774) were acquired from the PubChem database in the form of 3D SDF files (https://pubchem.ncbi.nlm.nih.gov/), which were subsequently used for docking purposes. For target enzymes, PDB ID: 5NN8(17) for α-glucosidase and PDB ID: 1CPU(18) for α-amylase were selected from the RCSB Protein Data Bank (PDB) and downloaded in the PDB file format. Missing residues in the 5NN8 crystal structure were completed with AlphaFold tools^19^ in ChimeraX v1.4.^10,20^ After the protein and ligand structures were obtained and prepared, they were submitted to the CB-Dock2 server. Visualization and analysis of protein–ligand interactions were performed using UCSF ChimeraX v.1.4 and BIOVIA Discovery Studio Visualizer v.21.

#### 2.6.2 Molecular Dynamics Simulations

Molecular dynamics (MD) simulations in this study were performed using Gromacs v.2021.2.^21^ The MD input files required for the simulation of protein–ligand complexes obtained from AutoDock Vina were prepared with the default settings of the CHARMM-GUI web server (https://www.charmm-gui.org/, accessed on 21.11.2022).^22^ AMBER99SB force fields^23^ were chosen to create topology files, which were also suitable for simulating protein–ligand complexes.^24^ One hundred fifty ns of MD simulation was run to 2 fs, and 1500 frames were recorded. MD trajectory root-mean-square deviation (RMSD) and H bond analysis were performed with gmx rms and gmx hbond scripts. Principal component analysis (PCA) was performed with gmx covar and gmx anaeig scripts. Binding free energy molecular mechanics Poisson–Boltzmann surface area (MMPBSA) measurements between the ligand and the protein were performed using gmx_MMPBSA tools.^25^ Trajectory plots were created with Grace-5.1.22, and MD animation videos were created with the PyMOL Molecular Graphics System v.2.4.1.

## 3 Results

### 3.1 α-Glucosidase, α-Amylase, and Tyrosinase Enzyme Inhibition Activity

The results of α-glucosidase and α-amylase and tyrosinase activities are described in Table 1. As a result, the CLM and CLE were found to be more active than others. It has been seen that the CLM extract was found to be even more active than acarbose in α-glucosidase (IC_50_ = 56.333 ± 0.986 μg/mL) and α-amylase (IC_50_ = 172.800 ± 0.816μg/mL) assays. Moreover, the CLE (IC_50_ = 204.067 ± 1.739 μg/mL) fraction also showed the highest inhibitory activity than acarbose in the α-amylase test. According to tyrosinase activity results, it has been seen that CLE (IC_50_ = 213.900 ± 1.553 μg/mL) fraction showed moderate activity compared with other extracts. According to the enzyme inhibitory results, the activities of the extract and fractions are statistically significant between each other and positive standards (*p* < 0.05).

**Table 1:** α-Glucosidase, α-Amylase, and Tyrosinase Results of *C. lycaonica* Extract and Fractions^a^

sample names	α-glucosidase assay IC_50_ (μg/mL)	α-amylase assay IC_50_ (μg/mL)	Tyrosinase assay IC_50_ (μg/mL)
CLM	56.333 ± 0.986^a^	172.800 ± 0.816^a^	261.667 ± 1.716^a^
CLP	396.033 ± 0.558^b^	437.767 ± 0.073^b^	565.192 ± 1.664^b^
CLE	435.833 ± 0.350^c^	204.067 ± 1.739^c^	213.900 ± 1.553^c^
CLB	-	960.600 ± 0.608^d^	245.301 ± 1.612^d^
CLW	-	310.767 ± 0.312^e^	332.622 ± 2.212^e^
acarbose	145.707 ± 0.451^d^	276.000 ± 0.205^f^	-
kojic acid			76.657 ± 1.370^f^

a Values are expressed as mean ± SD (*n* = 3). Values with the different letters within each column are significantly different (*p* < 0.05). “-“ not calculated. CLM: methanol extract of *C. lycaonica*, CLP: petroleum ether fraction of *C. lycaonica*, CLE: ethyl acetate fraction of *C. lycaonica*, CLB: *n*-butanol fraction of *C. lycaonica*, CLW: water fraction of *C. lycaonica*.

### 3.2 Total Phenolic Content (TPC) and Total Flavonoid Content (TFC)

The results of TPC and TFC contents of extract and fractions are given in Table 2. It was shown that among the extract and fractions, the CLM extract contains higher amounts of total phenolic compounds (283.168 ± 2.511 mg_GAE_/g_extract_) and total flavonoids (42.212 ± 1.411 mgCA/g_extract_) than others. According to the results, there was no statistically significant difference (*p* > 0.05) between the total contents of phenolic compounds of CLM extract and the CLP and CLS fractions. Moreover, the CLE fraction contained a high level of phenolic compounds following CLM extract (204.380 ± 2.473 mg_GAE_/g_extract_).

**Table 2:** Total Phenolic and Flavonoid Content and Antioxidant Activity Results of *C. lycaonica* Extract and Fractions^a^

samples	TPC (mg GAE/g_extract_)	TFC (mg CA/g_extract_)	FRAP (mmol Fe^2+^/g_extract_) (mmol Fe^2+/^g_Trolox_)	ABTS (μM Trolox/g_extract_) (μM Trolox/gBHA)
CLM	283.168 ± 2.511^a,b,e^	42.212 ± 1.411^a^	362.812 ± 2.291^a^	0.584 ± 0.023^a,c^
CLP	113.430 ± 1.204^b,d,e^	12.333 ± 2.517^b^	52.733 ± 1.201^b^	0.512 ± 0.053^b,e^
CLE	204.380 ± 2.473^c^	16.267 ± 1.079^c^	336.300 ± 1.045^c^	0.573 ± 0.003^c^
CLB	25.327 ± 1.213^d,e^	4.853 ± 2.179^d,e^	251.458 ± 1.471^d,e^	0.525 ± 0.012^d,e^
CLW	32.987 ± 1.410^e^	17.124 ± 1.258^e^	256.700 ± 1.809^e^	0.523 ± 0.004^e^
Trolox			6879.178 ± 0.745^f^	
BHA				0.605 ± 0.003^f^

a Values are expressed as mean ± SD (*n* = 3). Values are expressed as mean ± SD (*n* = 3). Values with the different letters within each column are significantly different (*p* < 0.05). CLM: methanol extract of *C. lycaonica*, CLP: petroleum ether fraction of *C. lycaonica*, CLE: ethyl acetate fraction of *C. lycaonica*, CLB: *n*-butanol fraction of *C. lycaonica*, CLW: water fraction of *C. lycaonica*.

### 3.3 *In Vitro* Antioxidant Activity

**Figure 3:**
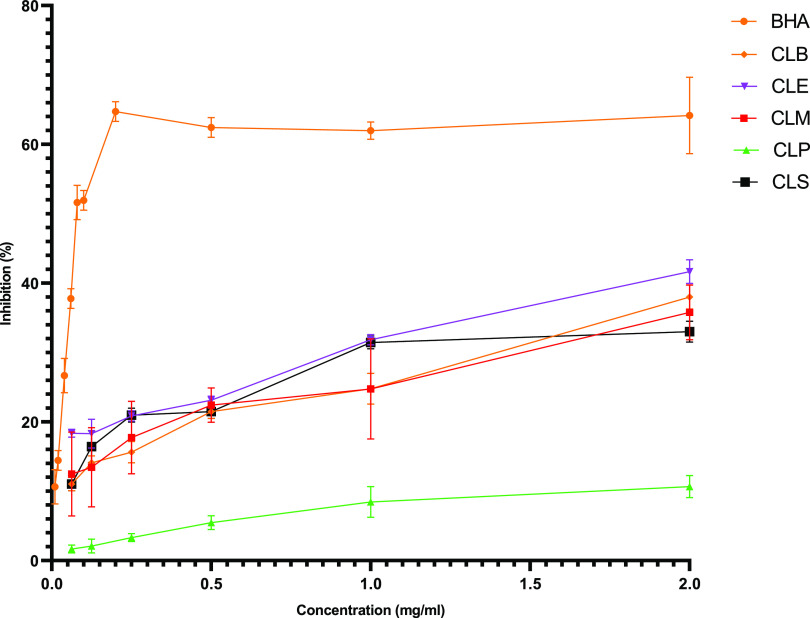
DPPH radical scavenging activity of *C. lycaonica* extract and fractions.

The results of antioxidant tests are given in Table 2 and Figure 3. The results showed that the extract and fractions demonstrated moderate DPPH activity. So, among the extract and fractions, the CLE fraction was more active than others, with 41% DPPH radical scavenging activity. This research used 0.5 mg/mL concentrations of extract, fractions, and BHA to determine ABTS activity. According to the results, the CLE (0.573 ± 0.003 Trolox/g_extract_) fraction was found to be the most active compared to others in this assay (*p* > 0.05). On the other hand, CLM extract (362.812 ± 2.291 mmol Fe^2+^/g_extract_) and CLE fraction were the most active (336.300 ± 1.045 mmol Fe^2+^/g_extract_) in the FRAP assay (*p* > 0.05).

### 3.4 Antimicrobial Activity

The MICs of the extracts and reference antimicrobials observed as the result of the study are presented in Table 3. The antimicrobial activities of the extracts were compared to reference antimicrobials. It was observed that the extract and fractions have moderate antimicrobial activity in the range of 128–256 μg/mL. Among the extract and fractions, CLM and CLE showed high antimicrobial activity against *C. albicans* and CLE fraction against *E. coli* microorganisms at 128 μg/mL. It has been demonstrated that extract and fractions’ antimicrobial activity is less than the reference antimicrobial agents.

**Table 3:** *In Vitro* MICs (μg/mL) Observed for the Extract, Fractions, and Reference Antimicrobial Drugs^a^

	microorganisms					
samples	S. a.	S. a.*	E. c.	E. c.*	C. a.	C. a.*
CLM	256	256	256	256	128	128
CLP	256	256	256	256	128	256
CLE	256	256	128	256	128	128
CLB	256	256	128	256	128	256
CLW	256	256	128	256	128	256
ampicillin	2	32	16	16		
gentamycin	1	8	1	16		
vancomycin	1	4				
fluconazole					1	1

a S.a.: *S. aureus* ATCC 29213; S.a.*: *S. aureus* isolate (MRSA); E.c.: *E. coli* ATCC 25922; E.c.*: *E. coli* isolate (contains broad spectrum β-lactamase enzyme – GSBL-); C.a: *C. albicans* ATCC 10231; C. a.*: *C. albicans* isolate.

Table 3 presents the extracts’ minimum inhibitory concentrations (MICs) and reference antimicrobials, as observed in the study. The antimicrobial activities of the extracts were compared to those of the reference antimicrobials. Results showed that the extract and fractions exhibited moderate antimicrobial activity in the 128–256 μg/mL range. CLM and CLE showed higher antimicrobial activity against *C. albicans*, while the CLE fraction showed high activity against *E. coli* at 128 μg/mL. However, the antimicrobial activity of the extract and fractions was not as potent as that of the reference antimicrobial agents.

### 3.5 LC-MS/MS Results

**Figure 4:**
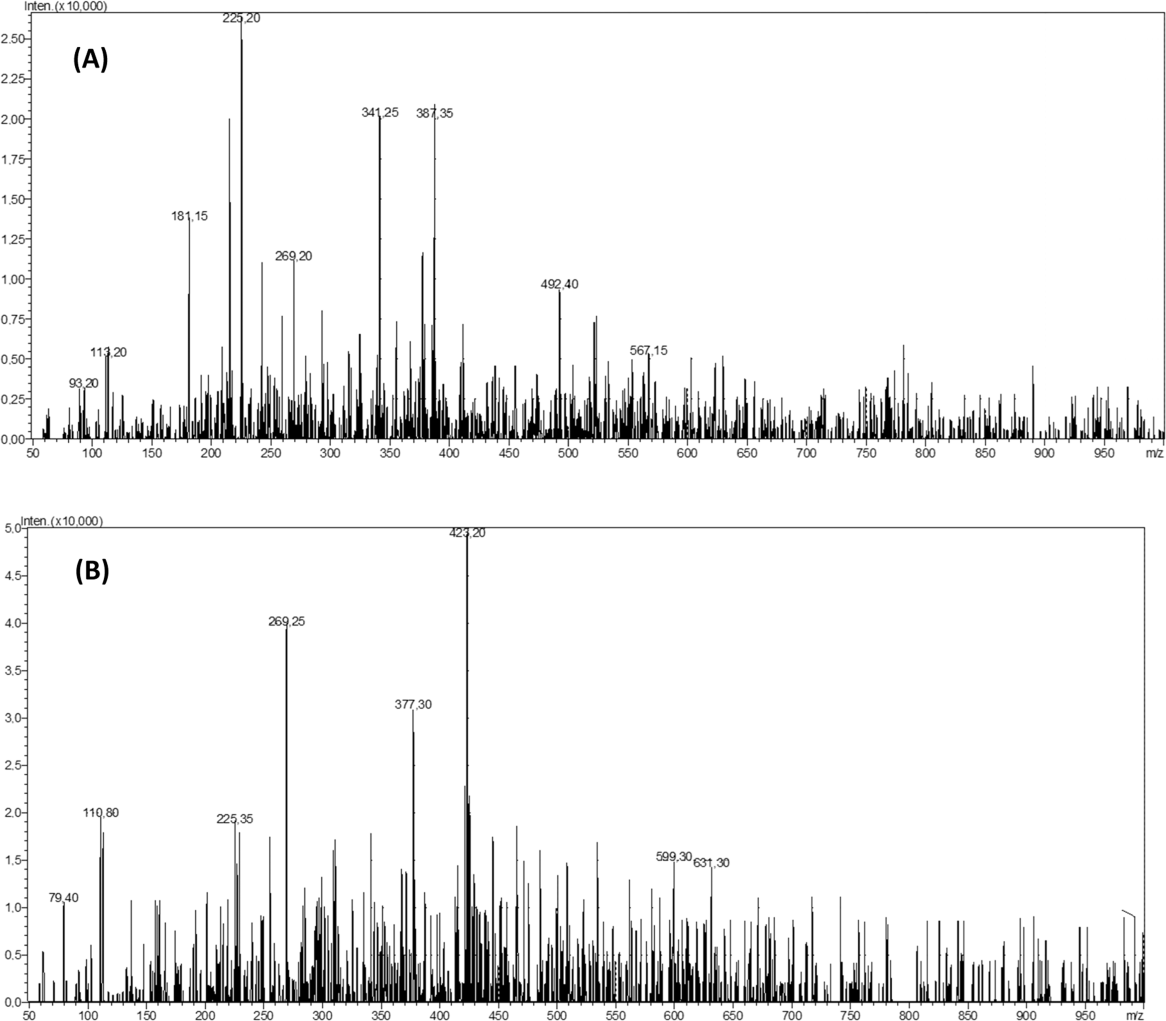
Mass spectra of the CLM extract (A) and the CLE fraction (B).

The bioactive compounds of active CLM extract and CLE fraction were determined by LC-MS/MS. The compounds were identified from registered mass spectral fragmentation patterns, the NIST (National Institute of Standards and Technology) mass spectral database (version 2.3), and literature data. According to the results, as myristoleic acid and apigenin were detected in both extract and fraction, caffeic acid-3-glucoside, geniposide, malvidin 3-galactoside, and phloretin 2′-xyloglucoside were determined in the CLM extract; and caffeic acid derivative, cinicin derivative, quercetin–hexose protocatechuic acid, and myrecitin-3-*O*-(2″-*O*-galloyl)-hexoside were determined in the CLE fraction only (Table 4 and Figure 4).

**Table 4:** Mass Spectral Characteristics and Identity of Compounds in CLM Extract and CLE Fraction

[M – H]^−^ (*m*/*z*)	MS/MS (*m*/*z*)	compounds	CLM	CLE	references
225	181, 165	myristoleic acid	+	+	(26)
269	117	apigenin	+	+	(26, 27)
341	179, 135	caffeic acid-3-glucoside	+	–	(26)
387	225, 191, 101	geniposide	+	–	(28)
492	254, 270, 285, 299, 313	malvidin 3-galactoside	+	–	(29)
567	435, 273	phloretin 2′-xyloglucoside	+	–	(30)
377	341, 215, 179, 161, 119	caffeic acid derivative-I	–	+	(31)
423	377, 359, 309, 263, 219, 201, 159, 131	cinicin derivative	–	+	(32)
599	463, 300	quercetin–galactoside protocatechuic acid	–	+	(30)
631	479, 317	myrecitin-3-*O*-(2″- *O*-galloyl)-glucoside	–	+	(30)

### 3.6 Molecular Docking

**Figure 5:**
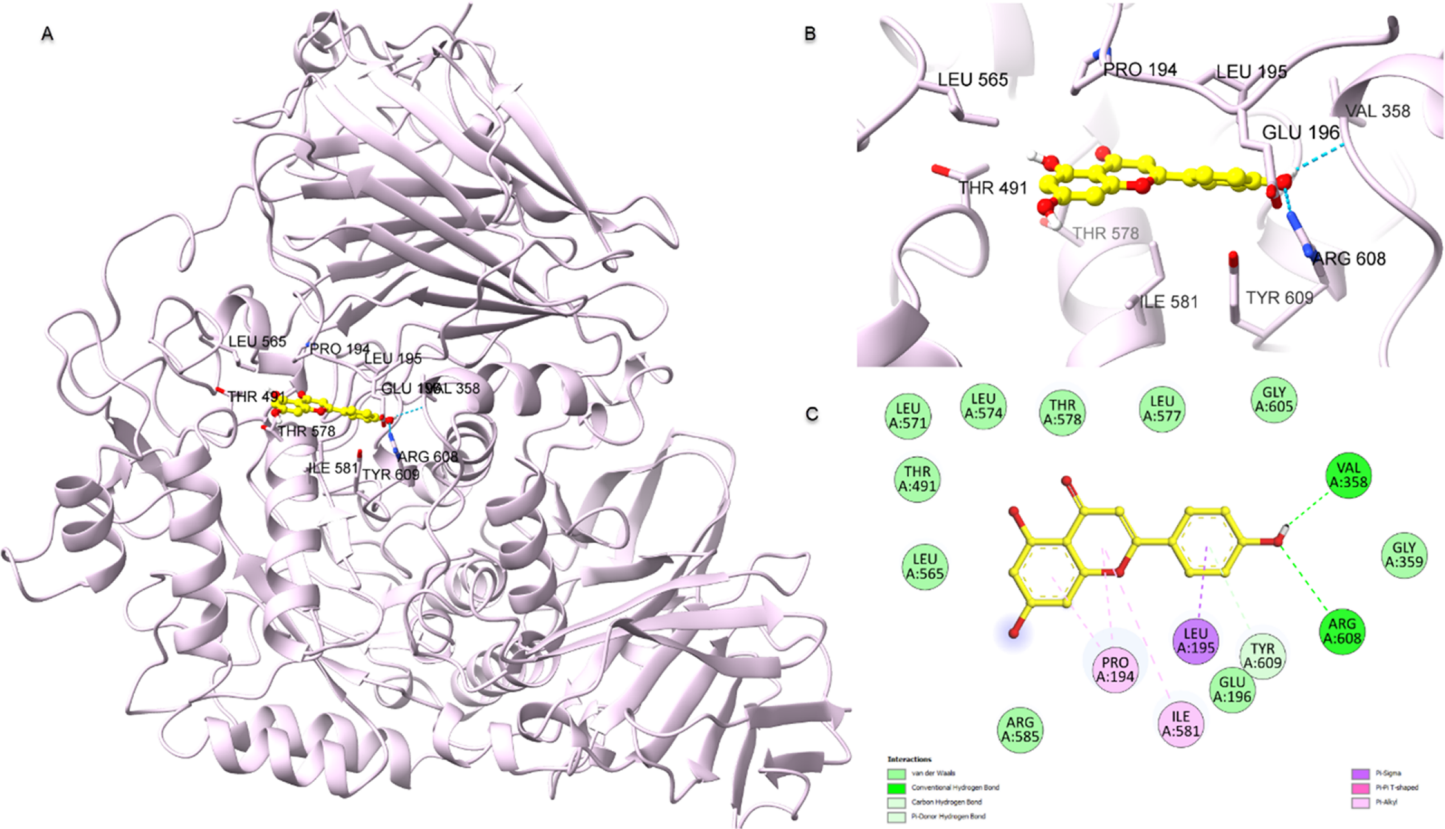
Molecular docking binding poses and protein–ligand interaction diagram of α-glucosidase with apigenin (PDB ID: 5NN8). (A) Full view of the α-glucosidase and apigenin complex. (B) Binding mode of apigenin at the α-glucosidase active site. (C) Schematic diagram of protein–ligand interaction depicting H bond and hydrophobic interactions.

**Figure 6:**
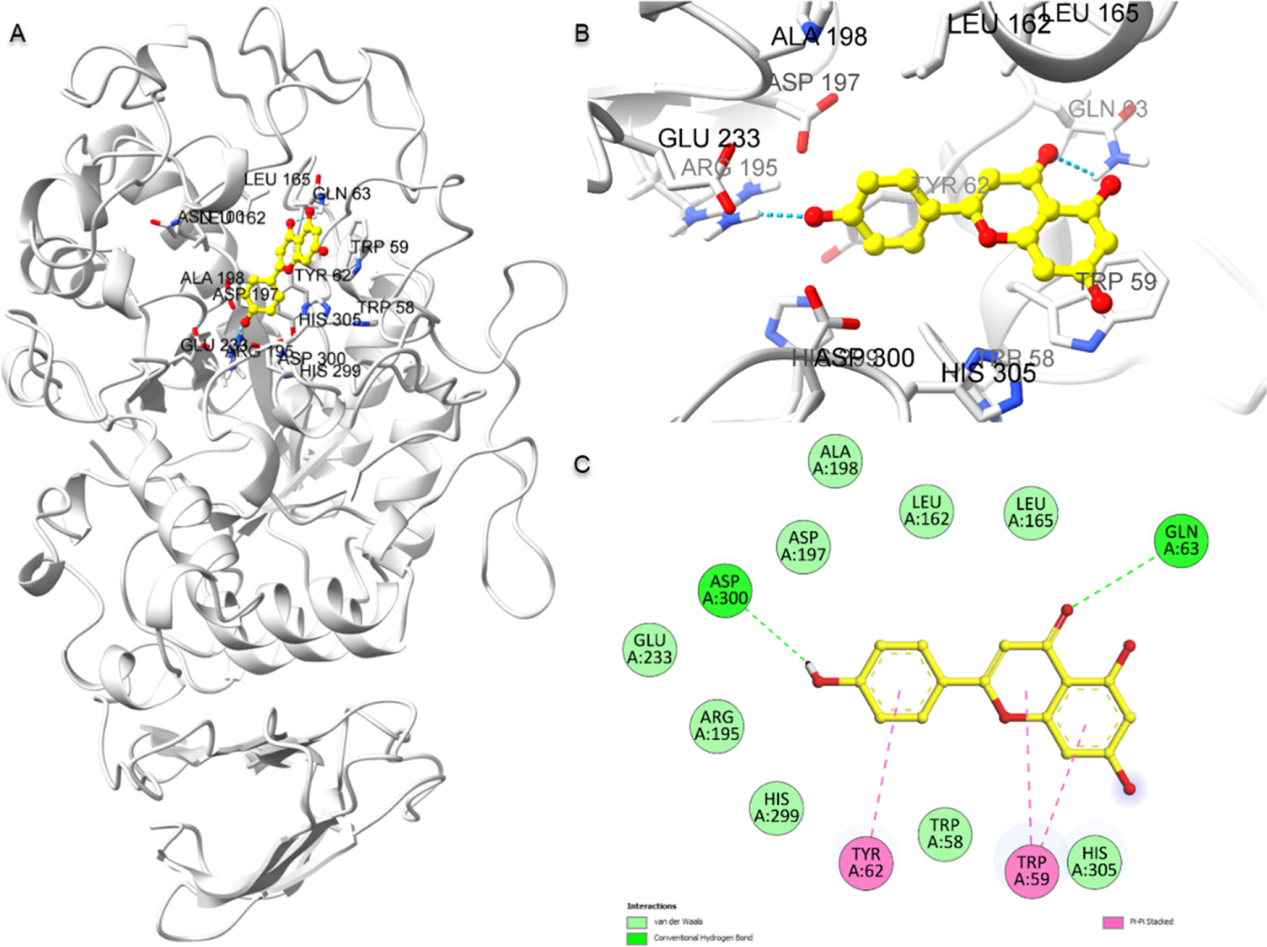
Molecular docking binding poses and protein–ligand interaction diagram of α-amylase with apigenin (PDB ID: 1CPU). (A) A complete view of the apigenin and α-amylase complex. (B) The manner in which apigenin attaches to the active sites of α-amylase. (C) A schematic diagram of protein–ligand interaction depicts H bonds and hydrophobic interactions.

A molecular modeling study of apigenin and myristoleic acid, common in active CLM and CLE extracts, was performed. The protein–ligand interaction energies and the residues with which apigenin, myristoleic acid, and acarbose formed with α-glucosidase and α-amylase are given in Table 5. The interaction energy of apigenin against both enzymes was lower than myristoleic acid and higher than acarbose. The interactions between apigenin and α-glucosidase in 2D and 3D are depicted in Figure 5. Apigenin formed two H bonds with key residues Arg608 (3.72 Å) and Val358 (4.46 Å) and π-sigma interactions with Leu195 (4.41 Å) at the α-glucosidase active site. The binding poses and protein–ligand interactions between the second target enzyme α-amylase and apigenin are shown in Figure 6. Apigenin formed one H bond with the critical amino acid Asp300 and van der Waals interactions with Glu233 in the active pocket of α-amylase. Also, another H bond with Gln63 produced π–π stacking interactions with Tyr62 (4.81 Å) and Trp59 (4.99 Å and 4.28 Å). Full details of protein–ligand interactions of myristoleic acid and acarbose are given in Table 5.

**Table 5:** Molecular Docking Results and Parameters of the Compounds Apigenin, Myristoleic Acid, and Reference Compounds Acarbose with Human α-Glucosidase and α-Amylase

	α-amylase (PDB ID: 1CPU)						α-glucosidase (PDB ID: 5NN8)					
					contact residues						contact residues	
compounds	Vina score	cavity volume (Å3)	center (*x*, *y*, *z*)	docking size (*x*, *y*, *z*)	H bonds	Van der Waals	Vina score	cavity volume (Å3)	center (*x*, *y*, *z*)	docking size (*x*, *y*, *z*)	H bonds	Van der Waals
apigenin	–8.6	859	5, 16, 44	30, 21, 21	Gln63 (4.19 Å) and Asp300 (4.81 Å)	Trp58, Trp59, Tyr62, Leu162, Thr163, Leu165, Arg195, Asp197, Glu233, His299 and His305	–6.9	745	–7, −20, 74	21, 21, 21	Val358 (4.46 Å) and Arg608 (3.72 Å)	Pro194, Leu195, Glu196, Val357, Gly359, Phe490, Thr491, Leu496, Leu565, Leu571, Leu574, Leu577, Thr578, Ile581, Gly605 and Tyr609
myristoleic acid	–4.8	859	5, 16, 44	30, 21, 21	Glu233 (4.88 Å)	Trp58, Trp59, Tyr62, His101, Tyr151, Leu162, Thr163, Leu165, Arg195, Asp197, Ala198, His201, Ile235, His299, Asp300 and His305	–4.4	745	–7, −20, 74	21, 21, 21		Pro194, Leu195, Glu196, Val357, Val358, Gly359, Phe490, Thr491, Leu496, Leu565, Leu571, Leu574, Leu577, Thr578, Ile581, Gly605, Arg608 and Tyr609
acarbose	–10.8	859	5, 16, 44	28, 28, 28	Arg195 (6.71 Å), Gln63 (5.49Å), Thr163 (4.86 Å), His299 (5.66 Å), Asp300 (5.26 Å) and His305 (4.03 Å)	Trp58, Trp59, Tyr62, Val98, His101, Gly104, Tyr151, Leu162, Gly164, Leu165, Asp197, Ala198, Lys200, His201, Glu233, Ile235, Glu240, Gly306 and Ala307	–8.5	745	–7, −20, 74	28, 28, 28	Asp356 (4.60 Å), Tyr360 (3.43 Å and 4.96 Å) and Arg608 (5.67 Å)	Glu196, Leu355, Gly359, Tyr360, Pro361, Phe362, Met363, Leu496, Ile581, His584, Arg585, Val588, Tyr609, His717 and Glu866

### 3.7 Molecular Dynamics (MD) Simulations

**Figure 7:**
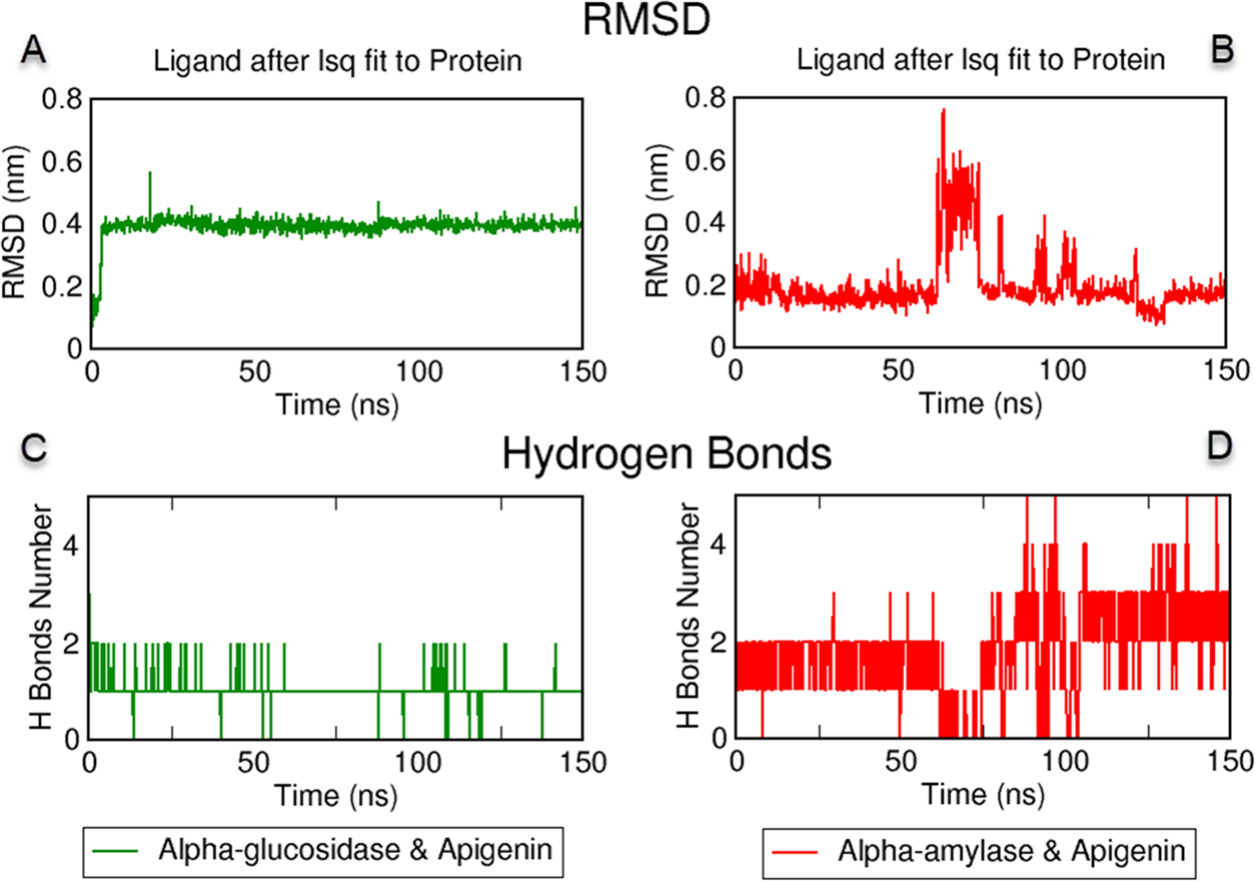
Molecular dynamics simulations trajectory analysis of the apigenin with α-glucosidase and α-amylase complex. (A) Root mean square deviation (RMSD) plot obtained by fitting apigenin to α-glucosidase enzyme, (B) RMSD plot obtained by fitting apigenin to α-amylase enzyme, (C) Intermolecular hydrogen bonds between apigenin and α-glucosidase, and (D) H bonds between apigenin and α-amylase for 150 ns.

MD simulation was performed for 150 ns in this study to demonstrate the stability of the apigenin and myristoleic acid complexes with α-glucosidase and α-amylase obtained from the molecular docking study.^33^ Fitting of apigenin to the backbone atoms of α-glucosidase and α-amylase, conformational changes of the ligand at the active site were analyzed by RMSD calculations.^34^ As shown in Figure 7A, the α-glucosidase active site of apigenin remained stable below 0.45 nm and had a mean value of 0.39 ± 0.39 nm. As given in Figure 7B in the other MD simulation, apigenin and α-amylase were stable around 0.2 nm for the first 60 ns, fluctuating up to 0.8 nm between 60 and 75 nm, after which there were shifts but below 0.4 nm, and remained stable on average around 0.2 nm. H-bond analysis is another vital trajectory analysis to examine protein–ligand stability.^35^ As shown in Figure 7C,D, fixed H bonds ranging from 1 to 2 over 150 ns usually form 2 H bonds up to 80 ns and usually 3 H bonds up to 150 ns between apigenin and α-amylase.

**Figure 8:**
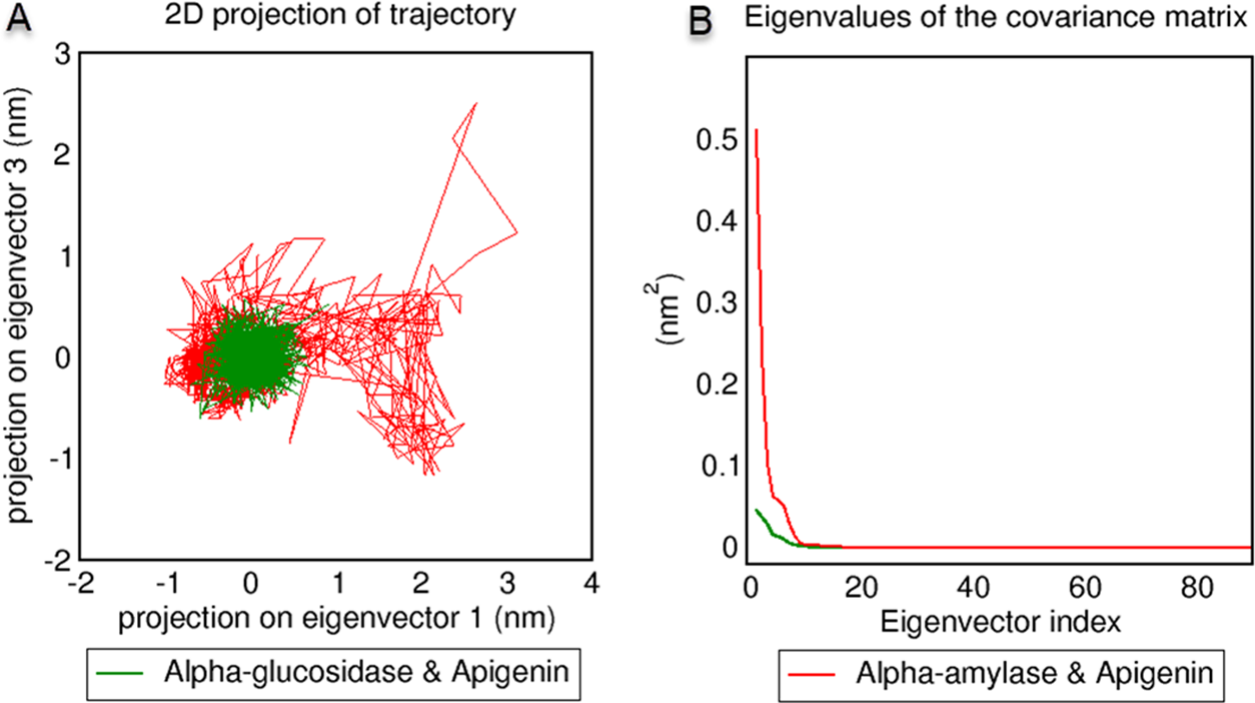
(A) Principal component analysis (PCA) 2D projection of apigenin atoms motion in α-glucosidase and to α-amylase was constructed by plotting the first two principal components (projection on eigenvector 1 and projection on eigenvector 3) in conformational space. (B) Eigenvalues plotted against the corresponding eigenvector indices obtained from the covariance matrix of the α-glucosidase and α-amylase backbone atoms fitted apigenin constructed from the equilibrated MD trajectories.

PCA analysis was performed to compare the binding stability of apigenin against α-glucosidase and α-amylase.^36^ As shown in Figure 8A, apigenin in the α-glucosidase–apigenin complex is between −0.7 and 0.9 nm in projection on eigenvector 1 and between −0.6 and 0.1 nm in projection on eigenvector 3. In contrast, in the α-amylase–apigenin complex, apigenin is between −1 and 3.2 nm in projection on eigenvector 1 and between −1.1 and 2.5 nm in projection on eigenvector 3. Other PCA analyses gave values below 0.05 and 0.5 nm^2^ with apigenin, α-glucosidase, and α-amylase according to the eigenvalues of the covariance matrix analysis, respectively (Figure 8B). As a final MD trajectory analysis, an MD animation video was created by recording binding poses at 100 frames between 0 and 150 to demonstrate the interactions between apigenin and α-glucosidase and α-amylase.^37^ The interactions of apigenin and α-glucosidase are given in Video S1, and the interactions of apigenin and α-amylase are shown in Video S2 (Supporting Information).

Binding free energy MMPBSA is an important method used to examine the stability and potency of the protein–ligand complex.^38^ Therefore, the MMPBSA between α-glucosidase and α-amylase with apigenin was calculated from 1500 frames between 0 and 150 ns.^39^ As detailed in Table 6, −28.39 ± 2.34 kcal/mol binding free energy between α-glucosidase and apigenin, and −22.93 ± 4.98 kcal/mol binding free energy between α-amylase and apigenin was measured. According to the MMPBSA calculation, apigenin gave higher binding energy and lower standard deviation values with α-glucosidase than with α-amylase.

**Table 6:** Binding Free Energy MMPBSA Computations of α-Glucosidase and α-Amylase with Apigenin from 1500 Frames between 0 and 150 ns^a^

	average energy (kcal/mol)	
energy component	α-glucosidase & apigenin	α-amylase & apigenin
ΔVDWAALS	–31.42 ± 2.56	–24.77 ± 2.97
ΔEEL	–13.58 ± 3.81	–28.51 ± 10.5
ΔEGB	21.12 ± 3.34	33.68 ± 6.92
ΔESURF	–4.52 ± 0.2	–3.33 ± 0.4
ΔGGAS	–44.99 ± 4.03	–53.28 ± 11.06
ΔGSOLV	16.6 ± 3.29	30.35 ± 6.63
ΔTOTAL	–28.39 ± 2.34	–22.93 ± 4.98

a Δ: Complex–Receptor–Ligand, VDWAALS: Van der Waals, EEL: Electrostatic energy, EGB: electrostatic solvation free energy evaluated from the generalized Born equation, ESURF: the nonpolar component of the solvation energy, GGAS: gas-phase energy, GSOLV: solvation free energy.

## 4 Discussion

The literature shows that few taxonomical studies on the *C. lycaonica* species exist. Various studies are related to inhibiting different enzymes and the antioxidant effect of the *Centaurea* species. So, this is the first study on enzyme inhibition, antimicrobial activities, antioxidant capacity, and phytochemical characterization of the methanol extract and different fractions.

In a study, the ethanol extract of the aerial parts of *C. rigida* was found to be active in the total antioxidant test (3.522 ± 0.166 mmol/L).^40^ In another study, the different extracts of the Turkish *Centaurea* species *C. hypoleuca* were studied in various activities. As a result, similar to *C. lycaonica* results, the flower ethyl acetate extract was more active than others in all activity tests, especially the antimicrobial activity assay (MIC value of 8 mg mL^–1^ against MRSA). Moreover, catechin and chlorogenic acid were detected as the major compounds in the extract.^41^ In another study, the ultrasonicated methanol extract of *C. amaena* exhibited high TPC and TFC, as well as total antioxidant, DPPH radical scavenging, and antimicrobial activity. Quercetin, quercetin-3-β-d-glucoside, and protocatechuic acid were detected as the main compounds of the extract.^42^ These data also supported the *C. lycaonica* results. According to another study, *C. baseri*, the local endemic species in Türkiye, was investigated. It has been shown that the methanol extract did not show antioxidant properties but was found to be the most active in antimicrobial tests against Candida utilis. The extract contained several biologically active compounds, including protocatechuic acid hexoside, apigenin, caffeic acid, ferulic acid, and cinicin derivative.^32^ The results of our study have agreed with this study, so the CLE fraction was found to be more active against Candida utilis, and the active compounds were also similar in this study. In a study, the enzyme inhibitory activity and antioxidant properties of *C. bornmuelleri* species were estimated. According to the results, the highest content of phenolic compounds and flavonoids was found in methanol and ethyl acetate extracts, such as *C. lycaonica*. Moreover, the water extract showed potent activity in DPPH (38.54 mg of TE/g_extract_) and ABTS (57.75 mg of TE/g_extract_) and FRAP assays (69.81 mg of TE/g_extract_) and ethyl acetate extract in tyrosinase (69.84 mg of kojic acid equivalent/g_extract_), α-amylase (19.90 mg of acarbose equivalent [ACAE]/g_extract_), and α-glucosidase (33.12 mg of ACAE/g_extract_) tests. Additionally, chlorogenic acid, luteolin derivatives, apigenin kaempferol-*O*-deoxyhexoside, and isorhamnetin were found at high concentrations in the extracts.^43^ Another study investigated the antioxidant, enzyme inhibitory, antimicrobial, and cytotoxic activities of *C. bingoelensi*. As a result, the highest phenolic content (41.57 mg of gallic acid equivalent (GAE)/g_extract_) was found in the hydro-methanol extract as well as the highest reducing capacity (136.87 and 82.16 mg of Trolox equivalent [TE]/g_extract_, for cupric reducing antioxidant capacity and ferric reducing antioxidant power, respectively), radical scavenging potential (70.72 and 76.53 mg TE/g_extract_, in DPPH and ABTS tests, respectively), and significant antifungal activity against *C. albicans*. Furthermore, phenolic acids and flavonoid derivatives were detected in the extract.^44^ Although, the endemic species from Türkiye *C. nerimaniae* also showed a potent antioxidant and antimicrobial capacity based on biologically active compounds such as cirsimaritin, hispidulin, apigenin, isokaempferide, and apigenin 7-*O*-glucoside.^45^ In another study, similar to *C. lycaonica*, the ethyl acetate fraction of the ethanol extract of C. virgata was found to be more active in DPPH (IC_50_ = 138.7 μg/mL) and ABTS radical scavenging activity. This fraction was also rich in phenolic compounds.^46^ In a study, C. drabifolia subsp. drabifolia and *C. lycopifolia* extracts were observed in terms of the TPC (18.33–32.84 mg_GAE_/g_extract_) and TFC content (2.88–22.39 mgRE/g_extract_) and antioxidant activity. Similarly to *C. lycaonica*, methanol, and water extracts showed more potent antioxidant abilities and enzyme inhibition effects (except for tyrosinase). In contrast to *C. lycaonica*, the water extract also exerted considerable antimicrobial effects.^47^ In another study, the ethyl acetate extract of the stem from *C. triumfetti* was found to have high antimicrobial and enzyme inhibition activity, as described in *C. lycaonica* results. Moreover, the major component of the extract was found to be chlorogenic acid.^48^ The acetone extract of *C. babylonica* showed the best antibacterial activity against *Bacillus cereus*, *P. aeruginosa*, and *C. albicans* (MIC: 1.6 mg/mL).^49^ A study determined the antioxidant capacities of *C. balsamita* and *C. albonitens* seed extracts by DPPH and ABTS assays. Results were 26.60 and 27.12% in DPPH and 80.61 and 95.99 mmol Trolox eq/g in ABTS, respectively. The total phenolic content of the seeds was determined to be 9019 and 11501 mg GAE/kg, respectively.^50^ In a study, various extracts of *C. pulcherrima* var. freynii were investigated. It was observed that the ethyl acetate extract showed the most effective activity, including total phenolic, flavonoid, antioxidant, and antimicrobial activities.^51^ As can be seen, these findings support the results of *C. lycaonica*. A study investigated the antioxidant activity of chloroform extracts prepared from the aerial parts of *C. kilaea*, *C. cuneifolia*, *C. salicifolia*, and *C. stenolepis*. Among all the tested extracts, the highest amounts of total phenolic and antioxidant capacity were found in the *C. salicifolia* extract.^52^

It is well known that the highest antidiabetic and antioxidant capacity is related to phenolic and flavonoid compounds.^53^ Based on these studies, it can be said that our results agreed with the findings described in the literature. Our study found the highest TPC and TFC content in the methanol extract and ethyl acetate fraction. With this, enzyme inhibition and antioxidant activities were also found in the methanol extract and ethyl acetate fraction. Moreover, it can be said that the detected compounds in CAM and CAE agreed with other reports on the *Centaurea* species.^54^ From molecular docking study results, we can consider that the potent antidiabetic and antioxidant activity of active extract and fraction may be explained by these compounds, especially apigenin and myristoleic acid.

A molecular docking study was carried out against α-glucosidase and α-amylase with apigenin and myristoleic acid, which are common in extract and fraction that are active and show higher activity than the standard compound. According to their interaction energies, apigenin showed a higher affinity for both targets than myristoleic acid. In addition, the interaction of apigenin with α-glucosidase was more potent than that of α-amylase, according to the binding energies. Apigenin formed critical H bond interactions necessary for activity with Arg608 at the α-glucosidase active site and Asp300 at the α-amylase active site.

According to the trajectory data and animation videos of 150 ns MD simulation to examine the stability between apigenin and α-glucosidase and between apigenin and α-amylase in an *in silico* physiological environment, apigenin formed potent interactions at both target protein active sites. However, according to RMSD, PCA analyses, and MMPBSA computations, the protein–ligand complex formed between apigenin and α-glucosidase was more stable than the protein–ligand complex formed between apigenin and α-amylase as *in vitro* enzyme assays. In addition, MD simulation of protein–ligand complexes formed between myristoleic acid and α-glucosidase and α-amylase was performed. Still, myristoleic acid did not form stable interactions with both target enzymes.

There are studies about the antidiabetic activity of apigenin in the literature.^55^ However, it is necessary to investigate whether the activity of the extract and fraction is due to these compounds or the synergistic effect of the substances.

## 5 Conclusions

The biological activity and phytochemical composition studies on the local endemic and unexplored species *C. lycaonica* was carried out for the first time in this study. This species’ methanol extract and ethyl acetate fraction showed potential enzyme inhibition and antioxidant activity. The phytochemical analyses of this extract and fraction showed the presence of phenolic compounds and flavonoids. These compounds’ molecular docking study results identified apigenin and myristoleic acid as active substances. However, further investigation is needed to determine the antidiabetic activity and the mechanism of action of these compounds.

## Data Availability

The data are contained within the article and the Supporting Information.

## Supporting Information Available

Video SI1: MD Simulation animation of 100 snapshots between 0 and 150 ns of human lysosomal acid-α-glucosidase with apigenin (MP4)
Video SI2**:** MD Simulation animation of 100 snapshots between 0 and 150 ns of human pancreatic α-amylase in complex with apigenin (MP4)

## Abbreviations

CLM: methanol extract of *C. lycaonica*
CLP: petroleum ether fraction of *C. lycaonica*
CLE: ethyl acetate fraction of *C. lycaonica*
CLB: *n*-butanol fraction of *C. lycaonica*
CLW: water fraction of *C. lycaonica*
DPPH: 1,1-diphenyl-2-picrylhydrazyl radical
ABTS: 2,2-azinobis (3-ethylbenzothiazoline-6-sulfonate) radical
FRAP: Ferric reducing ability power
TPC: the total phenolic content
TFC: total flavonoid content
LC-MS/MS: liquid chromatography–mass spectrometry/mass spectrometry
NIST: National Institute of Standards and Technology
